# Uptake of risk-reducing salpingo-oophorectomy in women carrying a *BRCA1* or *BRCA2* mutation: evidence for lower uptake in women affected by breast cancer and older women

**DOI:** 10.1038/bjc.2011.573

**Published:** 2011-12-20

**Authors:** L Sidon, S Ingham, T Clancy, R Clayton, A Clarke, E A Jones, F Lalloo, D G R Evans

**Affiliations:** 1 Department of Genetic Medicine, The University of Manchester, Manchester Academic Health Science Centre, Central Manchester University Hospitals Foundation Trust, St. Mary's Hospital, Manchester, UK; 2 Health Sciences, School of Community Based Medicine, Jean McFarlane Building, The University of Manchester, Oxford Road, Manchester, M13 9PL, UK; 3 Department of Gynaecology, Manchester Academic Health Science Centre, Central Manchester University Hospitals Foundation Trust, St. Mary's Hospital, Manchester, UK

**Keywords:** oophorectomy, *BRCA1*, *BRCA2*, uptake

## Abstract

**Background:**

Bilateral risk-reducing salpingo-oophorectomy (BRRSO) is the only effective way of reducing mortality from ovarian cancer. This study investigates uptake of BRRSO in 700 *BRCA1/2* mutation carriers from Greater Manchester.

**Methods:**

Dates of last follow-up and BRRSO were obtained, and the following variables were investigated: ovarian cancer risk/gene, age and breast cancer history. The date of the genetic mutation report was the initiation for Kaplan–Meier analysis.

**Results:**

The uptake of BRRSO in *BRCA1* mutation carriers was 54.5% (standard error 3.6%) at 5 years post testing compared with 45.5% (standard error 3.2%) in *BRCA2* mutation carriers (*P*=0.045). The 40–59 years category showed the greatest uptake for BRRSO and uptake was significantly lower in the over 60 s (*P*<0.0001). Of the unaffected *BRCA1* mutation carriers, 65% (standard error 5.1%) opted for surgery at 5 years post-testing compared with 41.1% (standard error 5.1%) in affected *BRCA1* mutation carriers (*P*=0.045).

**Conclusion:**

The uptake of BRRSO is lower in women previously affected by breast cancer and in older women. As there is no efficient method for early detection of ovarian cancer, uptake should ideally be greater. Counselling should be offered to ensure *BRCA1/2* mutation carriers make an informed decision about managing their ovarian cancer risk.

Mutations of the genes *BRCA1* and *BRCA2* are associated with a high lifetime risk of developing breast and/or ovarian cancer. Both *BRCA1* and *BRCA2* are tumour-suppressor genes. They have their own distinctive mechanisms of action and have a key role in maintaining genomic stability (Boulton, 2006). These genetic defects, however, only account for a small fraction of the overall breast and ovarian cancer rates. In the United Kingdom, *BRCA1* and *BRCA2* mutations were identified in 5.9% of women diagnosed with breast cancer under the age of 36 years, and in 4.1% of women diagnosed with breast cancer between the age of 36 and 45 years (Peto *et al*, 1999). Another two studies demonstrated that *BRCA1* and *BRCA2* mutations represent 10–15% of all ovarian cancers (Risch *et al*, 2001; Pal *et al*, 2005).

A large study carried out by five European centres between 1991 and 2007 assessed ovarian screening in 3532 women with increased ovarian cancer risk. The women were invited on a yearly basis for screening, which involved transvaginal ultrasound and serum Ca125 measurements. Kaplan–Meier analysis was used to compare survival rates between known *BRCA1/2* carriers and non-carriers. Carriers had a poorer survival with only 35% being alive at 10 years and it was concluded that screening ovarian cancer is currently ineffective (Evans *et al*, 2009b). Previous studies have assessed uptake of bilateral risk-reducing salpingo-oophorectomy (BRRSO) in unaffected women. These have demonstrated that uptake varies between countries (Metcalfe *et al*, 2008) and is dependent on cancer risk as well as age at the time of genetic testing (Evans *et al*, 2009c).

We undertook a study in the North West of England evaluating the rates of uptake of BRRSO among mutation carriers with comparisons between affected and unaffected mutation carriers and age groups.

## Methods

Families with a history of breast and ovarian cancer have been screened for *BRCA1/2* mutations (using a whole gene analysis including a test for large deletions) in the Manchester region since 1996. Women who attend the specialist genetic clinics with a family history of breast/ovarian cancer have a detailed family tree elicited with all first, second and if possible third degree relatives recorded. All cases of breast or abdominal cancers are confirmed by means of hospital/pathology records from the Regional Cancer Registries (data available from 1960) or from death certification. Initial mutation screening is usually carried out on an individual with either breast or ovarian cancer. Affected women in the past have had blood taken in their oncology clinic or at home after fairly minimal counselling. Both the genes have been tested with a whole-gene testing approach including multiple ligation-dependant probe amplification. Once a family-specific pathogenic *BRCA1/2* mutation is identified, predictive testing is offered to all known/traceable at-risk relatives. Pre-symptomatic testing usually involves at least two genetic counselling sessions before taking blood. Where possible all affected women with breast/ovarian cancer are tested to establish the true extent of *BRCA1/2* involvement in the family. Both affected and unaffected individuals are seen at a genetics clinic appointment and have a detailed discussion after receiving genetic test results. Mutation carriers are also offered follow-up via the genetic family register, with open access to the regional genetic service. They are contacted by post every 1–2 years to update information regarding screening and strategies to manage ovarian and breast cancer risk, and receive a newsletter. However, they are not seen in clinic on a regular basis.

All known female *BRCA1/2* mutation carriers were included in this study. Their details, those of all tested relatives and first-degree untested female relatives, were entered onto a Filemaker Pro 5 database (FileMaker, Inc., Santa Clara, CA, USA). Women who were being followed up elsewhere were excluded. Dates of last follow-up were obtained from the family files by looking at referral letters, clinic letters or recall for the genetic register letters. The annual recall letters have a specific section regarding ovarian screening and surgery, which facilitated the process. The dates of BRRSO were entered on the database and used to establish the proportion of women undergoing risk-reducing surgery. Women with ovarian cancer before learning their mutation status were excluded.

Once the data were collected, a Kaplan–Meier analysis was run commencing at the date of the genetic mutation report. This evaluated cumulative percentage uptake of risk-reducing surgery in *BRCA1* and *BRCA2* mutation carriers, comparing the two. Uptake among three different age groups was also assessed: the under 40, 40–59 and over 60 years. Uptake was also assessed for breast-cancer-affected female carriers compared with those unaffected at the time of their genetic test. Finally, the Cox proportional hazards model (StataMP11) was used to calculate the hazard ratio (HR), and looked at potential interactions between age, breast-cancer-affected females and those unaffected, *BRCA1*/2 status, and BRRSO. Statistical significance was inferred when the 95% confidence interval (CI) did not include unity.

## Results

Eight hundred and thirty-six women from 678 families with *BRCA1/2* mutations (359 *BRCA1*; 319 *BRCA2*) have tested positive for a *BRCA1* or *BRCA2* mutation in the Manchester genetics service. After excluding 104 women with ovarian cancer, 732 Manchester female patients were available for study. Thirty-two patients who provided a blood sample died before the mutation being identified, with eight (25%) having undergone BRRSO. These women were excluded from further analysis. Of the remaining 700 women, 386 were affected with breast cancer at the time of learning their genetic status, 309 had pre-symptomatic testing and 5 women were identified as obligate unaffected gene carriers. *BRCA2* carriers were, on average, older than *BRCA1* carriers both for pre-symptomatic and affected cases (Table 1). Eighty-nine women were further excluded from analysis as they had BRRSO before mutation finding (Table 1). The mean and median ages at mutation report was around 10 years younger for those unaffected with breast cancer (Table 1). Eighteen cases of ovarian cancer have occurred after genetic testing, four identified at the time of BRRSO and a further case of peritoneal primary cancer in a women having a non risk-reducing oophorectomy. The remaining 13 cases occurred in women who had not undergone BRRSO.

**Figure 1:**
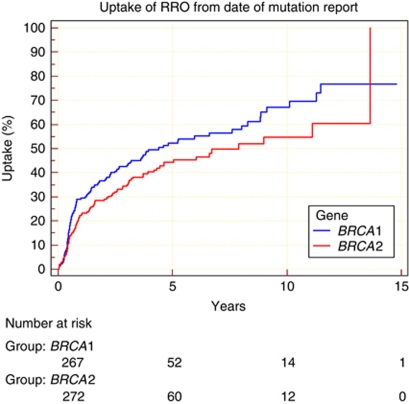
Comparison of cumulative percentage of BBRSO uptake in *BRCA1*/*2* mutation carriers from time of mutation report.

**Figure 2:**
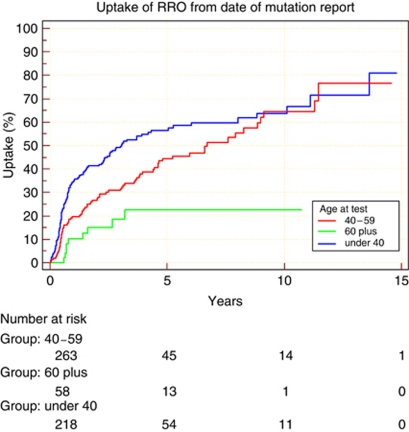
Comparison of cumulative percentage of BBRSO in three different age groups.

The uptake for BRRSO was greater in *BRCA1* mutation carriers 54.5% (standard error 3.6%) at 5 years post testing compared with 45.5% (standard error 3.2%) in *BRCA2* mutation carriers (Figure 1; *P*=0.045). Uptake was also shown to be the greatest in the 40–59 years of age category, as well as being quickest from time of mutation report. There was a delay in opting for surgery in the under 40 age group, but by 9 years uptake reached the same level (64%) as in the 40–59 age group. Uptake was significantly lower in women over 60 (Figure 2; *P*<0.0001). Of 65 women aged 60+ years at mutation report only 9 (14%) have since undergone surgery with an uptake of only 22% at 5 years. There are 42 (82%) women aged 60–69 years who retain their ovaries. None of 14 women aged 70+ have undertaken BRRSO. The Cox analysis confirmed this trend in uptake to BRRSO among the age groups, with women among the 40–59 age group more likely to opt for surgery than those <40 years (HR: 1.41; 95% CI: 0.98, 2.03). Conversely, women who were 60+years at mutation report followed the trend of being less likely to uptake to BRRSO than those <40 years (HR: 0.71; 95% CI: 0.34, 1.48). Neither of these results were statistically significant.

### Uptake in breast-cancer-affected *BRCA1*/2 mutation carriers compared with unaffected carriers

Of 386 women affected with breast cancer at mutation testing, 163 have had BRRSO (42%). Of these, 64 (39%) had their oophorectomy before the gene test results and were therefore removed from the further uptake analysis (29 *BRCA1* mutation carriers and 35 *BRCA2* mutation carriers). This can be compared with 314 unaffected women of whom 146 had BRRSO (46%) based on predictive testing. Of these, 24 (16%) had their ovaries removed before the date of mutation report.

**Figure 3:**
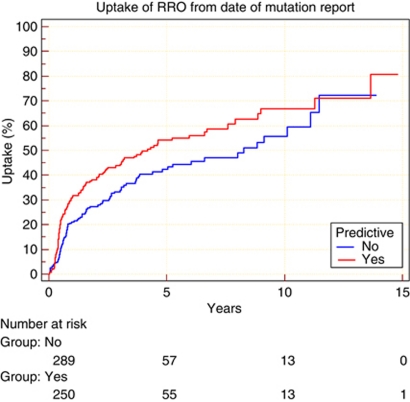
Comparison of cumulative percentage BRRSO from date of mutation report between breast-cancer-affected and unaffected carriers (the unaffected carriers had risk-reducing surgery based on predictive testing).

**Figure 4:**
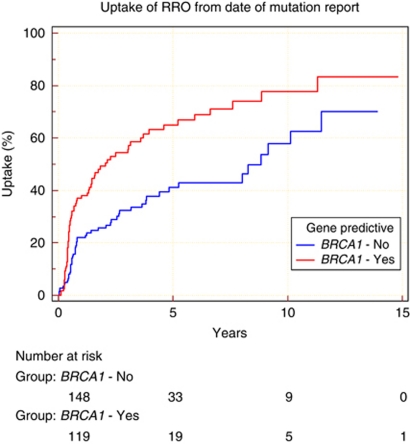
Comparison of cumulative percentage uptake of BRRSO in affected *BRCA1* mutation carriers with unaffected *BRCA1* mutations carriers.

The Kaplan–Meier analysis showed a statistically significantly lower uptake of BRRSO in breast-cancer-affected women compared with unaffected women (Figure 3; hazard ratio 0.7). This trend in BRRSO uptake in breast-cancer-affected women was supported using the cox hazard model (HR: 0.76; 95% CI: 0.53, 1.09) though this result was not statistically significant. The uptake for BRRSO was greater in unaffected mutation carriers 54.2% (standard error 3.6%) at 5 years post testing compared with 43.2% in affected carriers (*P*=0.0095). This difference was entirely attributable to lower uptake in affected *BRCA1* carriers (Figure 4; hazard ratio 0.48). The uptake for BRRSO was greater in unaffected *BRCA1* mutation carriers 65% (standard error 5.1%) at 5 years post testing compared with 41.1% (standard error 5.1%) in affected *BRCA1* mutation carriers (Figure 4; *P*=0.045). Cox proportional hazard analysis confirmed the trend that unaffected *BRCA1* carriers were over one and a half times more likely to undergo BRRSO than breast-cancer affected-*BRCA1* carriers (HR: 1.62; 95% CI: 1.01, 2.60). There was no statistically significant difference in uptake between affected and unaffected *BRCA2* carriers (HR: 1.0; *P*=0.97; HR: 0.97; 95% CI: 0.58, 1.63). There was also not any difference in uptake between affected *BRCA1 vs* affected *BRCA2* carriers (HR: 0.92; *P*=0.68). Therefore, differences between *BRCA1* and *BRCA2* can be explained by the higher uptake of BRRSO in women undergoing pre-symptomatic testing for *BRCA1* mutations; 65% (standard error 5.1%) at 5 years post testing compared with 45% of those undergoing pre-symptomatic testing for *BRCA2* mutations (HR: 1.8; *P*=0.0008).

Eighteen patients (14 *BRCA1*; 4 *BRCA2*; age range 37–73 years, median 58) have developed ovarian cancer after receiving their positive mutation test results. Four of these malignancies were present at the time of BRRSO (three: stage 1; one: stage 3) and two of these women have subsequently died. A further case of primary peritoneal cancer occurred in a women who had had a non-risk-reducing full vaginal hysterectomy and BSO 6 years before; she also died. Thirteen further cases occurred in women undergoing screening with annual transvaginal ultrasound and 4–12 monthly CA125 blood testing. One was at stage 1, two were at stage 2; eight at stage 3 and one at stage 4. Six of the thirteen cases have subsequently died.

## Discussion

This study has shown that uptake of BRRSO is dependent on several factors: ovarian cancer risk, the genetic cause, age and previous personal history of breast cancer. The uptake of surgery was related to risk of developing ovarian cancer as demonstrated by a significantly higher uptake in *BRCA1* mutation carriers, who have a lifetime ovarian cancer risk of 39–65%, compared with *BRCA2* mutation carriers, whose lifetime risks are 10–37%. However, this is the first time that differences in uptake of BRRSO between *BRCA1* and *BRCA2* have been shown to be an effect completely explained by higher rates of uptake in unaffected *BRCA1* carriers. We have previously reported the difference between those undergoing pre-symptomatic testing (Evans *et al*, 2009c), but there has been no difference in uptake among *BRCA1/2* affected individuals. It is possible that the improved adjuvant options such as use of aromatase inhibitors in *BRCA2* carriers, who have predominantly suffered from ER-positive breast cancers, could have equalised the effect of higher risk of ovarian cancer in *BRCA1* carriers. Women may have had advice from their oncologists in this respect, but overall the reasons for this remain unclear.

Early oophorectomy does have its problems with increased menopausal symptoms, (Madalinska *et al*, 2005; Challberg *et al*, 2011) and an increased risk of osteoporosis (Madalinska *et al*, 2005) and heart disease. However, this can be limited by careful use of HRT until at least 50 years of age in those without prior breast cancer (Howell and Evans, 2011). Nonetheless, the risks of an ovarian type cancer are very substantially reduced with BRRSO (Kauff *et al*, 2002, 2008; Rebbeck *et al*, 2002; Evans *et al*, 2009a), and although peritoneal primary cancer does occur this may be less common if surgery is undertaken in a careful way to remove all ovarian cells possible (Evans *et al*, 2009a).

It is not surprising that uptake is delayed in the under 40 age group as it is likely that these younger women may wish to retain their ovaries until their families are complete. In addition, the risk of ovarian cancer remains relatively low until after the age of 40. However, the uptake rate eventually catches up with that in the 40–59 age category. The over 60 age group has a substantially lower uptake rate, and elucidating the reasons for this is beyond the scope of this study. It may be due to the perception that if they have not developed cancer yet they will not develop it in the future. This is certainly anecdotally something we have heard from older women in clinic. Although older women will have a residual absolute risk that is lower than in younger women, they still have significant risks. We have shown that 33% of the ovarian cancers developing after testing occurred in this age group despite them only accounting for 9% of the 612 women who had not undergone BRRSO before testing. Given that all these women are post menopausal, they should still be encouraged to think seriously about BRRSO if they have reasonable life expectancy and are fit enough to undergo surgery.

Uptake of BRRSO in breast-cancer-affected women was shown to be lower than in unaffected women. However, again this difference is explained by higher uptake in *BRCA1* carriers who have undergone pre-symptomatic testing not by any differences in uptake in *BRCA2*. To our knowledge, this is the first study to describe such a trend and this finding should be investigated further. Both the sets of mutation carriers have better survival with their breast cancers after oophorectomy (Moller *et al*, 2002). The lower overall uptake in affected individuals raises the possibility that there may be differences in the counselling that these women have received. Women undergoing pre-symptomatic testing usually have 3–4 appointments including their session to receive their *BRCA1/2* results. However, affected women may only receive a single session from the genetics department to discuss the implications of being a carrier on ovarian cancer risks and their options in managing that risk. This approach is typical in the United Kingdom, although our register does offer more follow-up than most clinics are able to provide. Nonetheless several clinics in the London area now offer a ‘carrier’ clinic with patients invited to clinic for ongoing assessment (Bancroft *et al*, 2010). This highlights the potential importance of regular genetic counselling appointments regarding risk-reducing options in women who carry a *BRCA1* or *BRCA2* mutation, although we do not know whether uptake of BRRSO is higher in the carrier clinic centres than in those that do not use this model. As a result of the findings in the present study, we are now offering at least one further follow-up clinic appointment after receiving results to enable a further discussion of the options available.

## Overall conclusions

Unfortunately no effective screening programme against ovarian cancer has been devised to date. However, BRRSO in *BRCA1* and *BRCA2* mutations carriers has proven to be effective in reducing the rates of ovarian cancer as well as reducing the rates of breast cancer in the under 50 age group. These patients should therefore be encouraged to undergo BRRSO in the context of a discussion of risks and benefits. The results of the present study demonstrate a lack of uptake of risk-reducing surgery in women over 60 years of age and in women who have previously had breast cancer. As some of these women go on to develop ovarian cancer, it is important that they are fully informed of the risks of retaining their ovaries, and ideally the rates of uptake should be greater. It is therefore important to ensure regular follow-ups and counselling regarding surgical options to potentially reduce BRCA-related cancer mortality.

## Figures and Tables

**Table 1:** Proportion of pre-symptomatic and diagnostic tests among *BRCA1/2* carriers and uptake of BRRSO

	**Affected *BRCA1***	**Affected *BRCA2***	**Unaffected *BRCA1***	**Unaffected *BRCA2***
Number	198	188	157	157
Age range at mutation report (mean; median)	24–84 (47.56; 46.37)	26–88 (51.09; 50.22)	18–64 (37.64; 37.5)	19–68 (40.7; 42.33)
Number having BRRSO before mutation finding	29/198 (14.5%)	35/188 (18.6%)	15/157 (9.5%)	10/157 (6.4%)
Proportion who have so far undergone surgery after mutation finding	52/169 (30.7%)	47/153 (30.7%)	70/142 (49%)	51/147 (35%)
Time in years from mutation test to BRRSO: range (mean; median)	0.1–11.45 (2.29; 0.78)	0.1–11.1 (1.77; 1.05)	0.1–11.28 (1.63; 0.58)	0.1–8.98 (1.75; 0.86)

Abbreviation: BRRSO=bilateral risk-reducing salpingo-oophorectomy.
